# Human carbonic anhydrases and post-translational modifications: a hidden world possibly affecting protein properties and functions

**DOI:** 10.1080/14756366.2020.1781846

**Published:** 2020-07-10

**Authors:** Anna Di Fiore, Claudiu T. Supuran, Andrea Scaloni, Giuseppina De Simone

**Affiliations:** a Istituto di Biostrutture e Bioimmagini-National Research Council, Napoli, Italy; b NEUROFARBA Department, Pharmaceutical and Nutraceutical Section, University of Firenze, Sesto Fiorentino, Italy; c Proteomics and Mass Spectrometry Laboratory, ISPAAM, National Research Council, Napoli, Italy

**Keywords:** Carbonic anhydrase, post-translational modifications, proteomics

## Abstract

Human carbonic anhydrases (CAs) have become a well-recognized target for the design of inhibitors and activators with biomedical applications. Accordingly, an enormous amount of literature is available on their biochemical, functional and structural aspects. Nevertheless post-translational modifications (PTMs) occurring on these enzymes and their functional implications have been poorly investigated so far. To fill this gap, in this review we have analysed all PTMs occurring on human CAs, as deriving from the search in dedicated databases, showing a widespread occurrence of modification events in this enzyme family. By combining these data with sequence alignments, inspection of 3 D structures and available literature, we have summarised the possible functional implications of these PTMs. Although in some cases a clear correlation between a specific PTM and the CA function has been highlighted, many modification events still deserve further dedicated studies.

## Introduction

**Figure 1.**
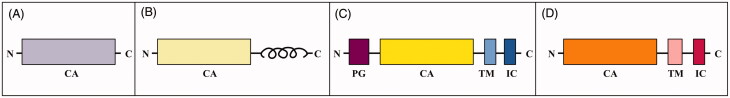
Schematic representation of (A) CAs containing only of the catalytic domain, (B) CA IV, (C) CA IX and (D) transmembrane isoforms CA XII and CA XIV.

Human carbonic anhydrases (CAs) are zinc-containing enzymes that catalyse a simple but fundamental physiological reaction, *i.e.* the reversible hydration of carbon dioxide to bicarbonate ion and proton^1^. Consequently, these enzymes are involved in several important patho-physiological processes related to respiration, transport of carbon dioxide between metabolising tissues and lungs, pH homeostasis, electrolyte secretion in various tissues/organs as well as biosynthetic reactions^1–3^. Fifteen isoforms have been identified so far, among which only twelve are catalytically active (CA I–IV, VA–VB, VI–VII, IX, and XII–XIV), whereas three are devoid of catalytic activity and named CA-related proteins (CARP VIII, X, and XI)^2^. Besides differing in catalytic activity, oligomerization pattern, tissue expression and subcellular localisation, these enzymes present a diverse domain organisation (Figure 1). Indeed, cytosolic (CA I, II, III, VII and XIII, and CARP VIII, X and XI), mitochondrial (CA VA and VB) and secreted (CA VI) isoforms consist of a unique domain hereafter indicated as CA domain (Figure 1(A)). In addition to the CA domain, the transmembrane isoforms (CA IX, CA XII and CA XIV) have a transmembrane (TM) region and an intracellular (IC) tail^4^ Figures 1(C) and 1(D); CA IX also contains an additional extracellular portion, known as proteoglycan-like (PG) domain^5^ (Figure 1(C)). Finally, CA IV presents a short segment at the C-terminus that is anchored to the cell membrane (Figure 1(B))^6^.

A large number of structural studies allowed to elucidate the CA domain three-dimensional arrangement for most isoforms^1^, showing that, as expected on the basis of their high sequence homology, these domains present a similar structure, which is characterised by a central twisted mainly antiparallel β-sheet surrounded by helical connections and additional β-strands. In the catalytically active isoforms, the active site is located in a wide and deep cavity, which spans from the protein surface to the centre of the molecule. The catalytic zinc ion is located at the bottom of this cavity, tetrahedrally coordinated by three conserved H residues and a water molecule/hydroxide ion^1^.

The CA-catalysed reaction follows a two-step mechanism described by Equations (1) and (2)^1^.

(1) $${\text{EZn}}^{\text{2}+}-{\text{OH}}^{-}+{\text{ CO}}_{\text{2}}⇆{\text{EZn}}^{\text{2}+}-{\text{HCO}}_{3}^{-}⇆\overset{{\text{H}}_{\text{2}}\text{O}}{{\text{EZn}}^{\text{2}+}}-{\text{H}}_{\text{2}}\text{O }+{\text{ HCO}}_{3}^{-}$$

(2) $${\text{EZn}}^{\text{2}+}-{\text{H}}_{\text{2}}\text{O}+\text{B}⇄{\text{EZn}}^{\text{2}+}-{\text{OH}}^{-}+{\text{BH}}^{+}$$

In the first step, the Zn^2+^-bound hydroxide leads a nucleophilic attack to CO_2_ resulting in the formation of HCO_3_^−^, which is then replaced by a water molecule, with the generation of the catalytically inactive form of the enzyme EZn^2+^−H_2_O (Equation (1)). In the second step (Equation (2)), which is the rate-limiting one, the zinc-bound hydroxide is regenerated through a proton transfer reaction from the zinc-coordinated water molecule to the bulk solvent (B)^7–10^. In the majority of the isoforms, a residue placed in the middle of the active site cavity, generally an H residue, assists this step acting as a proton shuttle^11^^,^^12^.

Notwithstanding the fact that a huge amount of literature is available on biochemical, functional and structural aspects of CA family, the occurrence and the functional implications of protein post-translational modifications (PTMs) have been poorly investigated so far. The application of different proteomic approaches in PTM-centred studies has generated large datasets of modification information, which have demonstrated the occurrence of thousands of modified proteins in human biological tissues/body fluids, whose nature and modified amino acids have been catalogued in dedicated (but often neglected) public databases^13–42^. These investigations have provided important, but fragmentary information on CAs, whose relevance has never been considered in its entirety. In order to fill this gap, here we have carried out a human CA-centred search in PTM databases revealing an unexpected record of experimentally validated modification data on these proteins. We have combined resulting data with sequence alignment analysis, allowing the recognition of frequent modifications at specific amino acids, conserved in various CAs. For each CA isoform, except CA VA, CA VB, CARP X and CARP XI, whose CA domain has not been structurally characterised yet, identified PTMs have been assigned on the corresponding three-dimensional structure. With the exception of disulphide bridges and other specific cases, the frequent recognition of modified residues on the enzyme surface has suggested the expected, prevalent occurrence of PTM-generating reactions on corresponding folded molecules. In other negligible cases, modified amino acids have been identified within internal regions of the protein structure, highlighting either the presence of highly reactive residues therein or the possible occurrence of above-mentioned PTM reactions on partially unfolded and/or degraded components. In some cases, modifications affected amino acids essential for enzyme catalysis and molecular stability, with a possible impact on corresponding protein function. All these data are discussed here with respect to information from the available literature on both the catalytic mechanism of each CA isoform and the corresponding, specific role in important human patho-physiological processes.

## Analysis of PTMs detected in human CA isoforms

**Figure 2.**
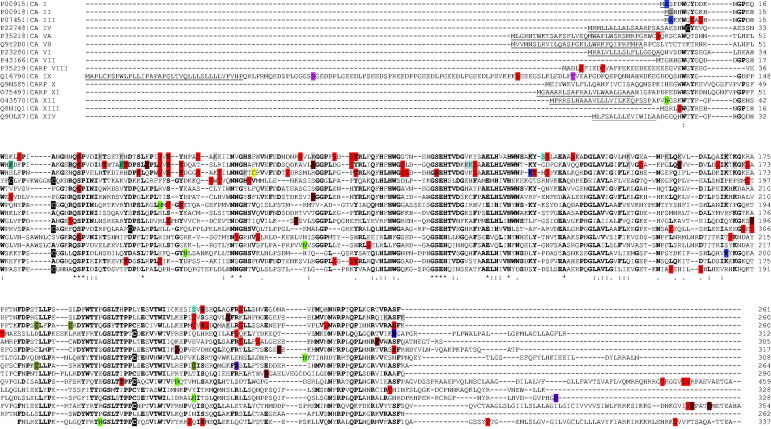
Sequence alignment of various human CA isoforms. Underlined are the signal peptide portions. In bold are reported identical residues at the same position present in at least seven isoforms. With asterisk are reported identical residues at the same position present in all isoforms. With colon are reported residues at the same position showing a high homology in CA isoforms. With dot are reported residues at the same position showing a low homology in CA isoforms. N-terminal residues subjected to both acetylation and phosphorylation are highlighted in dark grey. S, T and Y residues subjected to phosphorylation are highlighted in red. S and T residues subjected to both phosphorylation and O-glycosylation are highlighted in light blue. S and T residues subjected to O-glycosylation are highlighted in pink. C residues involved in disulphide bonds, subjected to S-glutathionylation and S-nitrosylation are highlighted in black, rotten green and yellow, respectively. K residues subjected to non-enzymatic (NE) glycation, acetylation and ubiquitinylation are highlighted in light grey, dark blue and brown, respectively. K residues subjected to both non-enzymatic glycation and acetylation are highlighted in light blue. K residues subjected to both acetylation and ubiquitinylation are highlighted in sugar paper colour. R residues subjected to methylation are highlighted in purple. N residues subjected to N-glycosylation are highlighted in green. S residues subjected to GPI anchoring are highlighted in dark blue.

**Figure 3.**
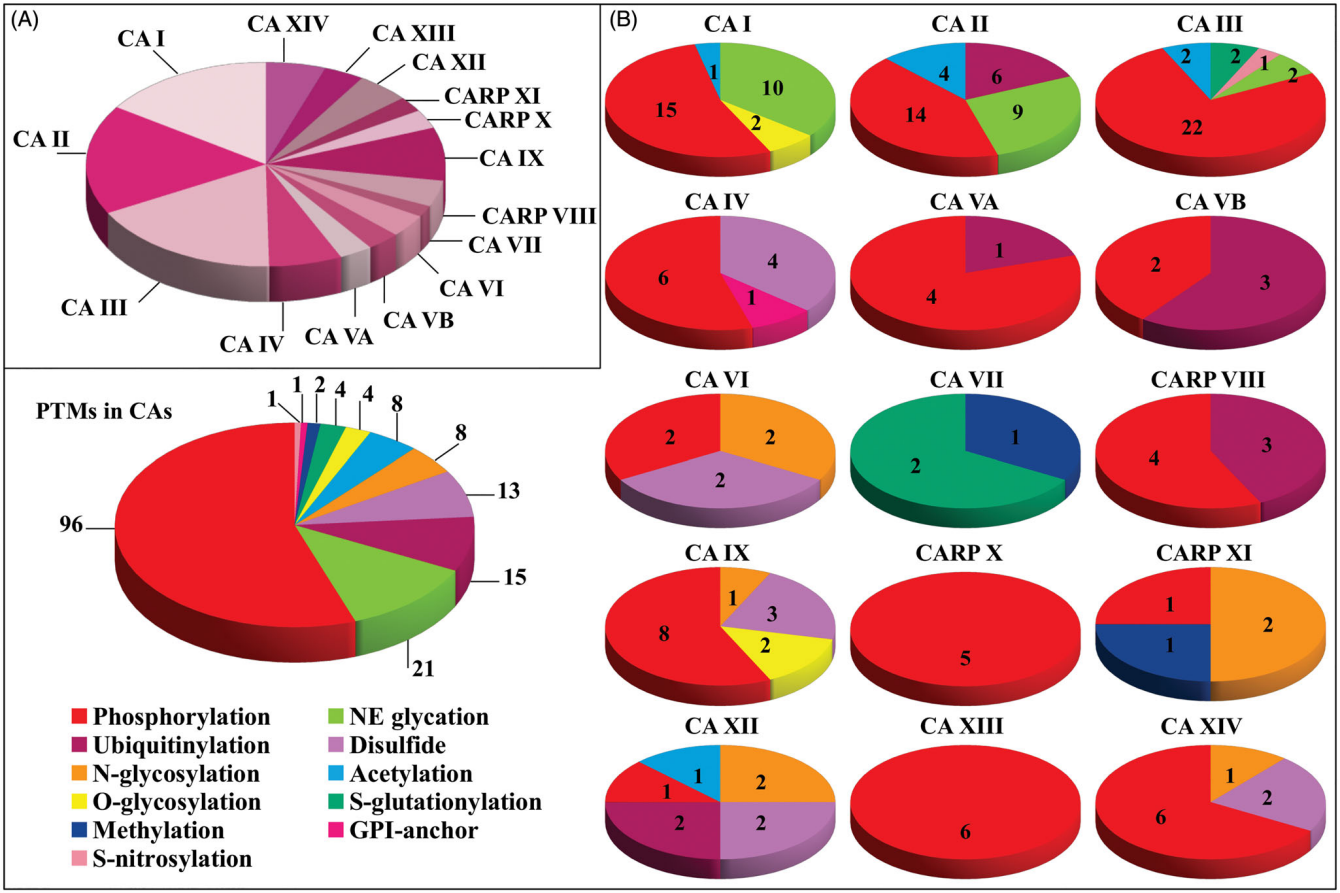
Pie chart representation reporting (A) the number of modified amino acids in the different CA isoforms, and (B) the nature and the number of the modifications present in each CA isoform and in the whole CA family.

With the aim to identify modified amino acids present in human CA isoforms, as described in available PTM databases and in the supplementary material of corresponding studies, a whole of 30 protein PTM repositories were searched in parallel. These databases contained information on more than 60 PTMs present in human proteins. Some of these repositories were centred on specific modifications. For example, PhosphoSitePlus^15^, qPhos^16^, Phospho.ELM^17^, PhosphoNET^13^, dbPAF^18^, LymPhos2^19^, PhosphoPep2^20^, and PepCyber^21^ contained only phosphorylation data; UbiProt^22^ and mUbiSiDa^23^ included ubiquitinylation results; Unipep^24^ contained information on N-linked glycosylation sites; O-GlycBase6^25^ was dedicated to O-glycosylation data; SwissPalm2^26^ was focussed on data on protein S-palmitoylation; GPI-AP^27^ included glycosylphosphatidylinisotol (GPI)-anchored protein information; dbGSH^14^ and dbSNO^28^ were dedicated to S-glutathionylation and S-nitrosylation data, respectively; PRENbase^29^ contained information on farnesylated and geranylated amino acids in proteins. Conversely, other databases contained information on multiple PTMs, such as UniProtKB^30^, PHOSIDA^31^, NetXProt^32^, PLMD^33^, iPTMNet^34^, RedoxDB^35^, CarbonylDB^36^, HPRD^37^, ProteomeScout^38^, ActiveDriverDB’20^39^, TopPTM^40^, CPLM^41^, and DeepNitro^42^. In some cases, these modification inventories reported results from the same studies, and thus provided redundant data. Final information was collected on July 2019 and was processed later on. This CA-centred analysis revealed the occurrence in human isoforms of phosphorylated, acetylated, ubiquitinylated, methylated, N-glycosylated, O-glycosylated, S-glutathionylated, S-nitrosylated, disulphide-linked, non-enzymatically glycated and GPI-modified amino acids at specific sequence positions. Results are summarised in Figure 2. In particular, 26, 29, 29, 11, 5, 5, 6, 3, 7, 14, 5, 4, 8, 6 and 9 modified residues were overall identified in CA I, CA II, CA III, CA IV, CA VA, CA VB, CA VI, CA VII, CARP VIII, CA IX, CARP X, CARP XI, CA XII, CA XIII and CA XIV, respectively (Figure 3(A)). In few cases, the concomitant occurrence of phosphorylation/O-glycosylation (at the same S residue) or acetylation/ubiquitinylation/glycation (at the same K residue) was observed. Collected data summarised the whole information from cell lines and biological tissues/body fluids described in proteomic studies and, as expected, also reflected the abundance of different CAs in the whole set of investigated human samples. The distribution of the different PTM types present in each CA and in all human family is reported in Figure 3(B). Besides to highlight the most frequent modifications in each isoform, these results demonstrated that the PTM representation in the protein family follows the following order: phosphorylation, non-enzymatic glycation, ubiquitinylation, disulphide formation, N-linked glycosylation and acetylation, S-glutathionylation, O-glycosylation, and others.

When the occurrence of PTMs was observed in the context of the whole CA family, after alignment of all isoforms, some conserved residues emerged as adducted in various isozymes, suggesting a possible functional role of the corresponding modification. Thus, the position of the modified residue on the three-dimensional structure was analysed, with the aim to verify if it was either on the protein surface or in the corresponding inner core. The close proximity of each PTM to the protein catalytic residues was also evaluated in order to investigate on an eventual regulatory role of it on the enzyme mechanism.

In the following sections, results obtained from this analysis will be discussed according to each type of PTM. Amino acid numbering utilised here corresponds to that used in UniProtKB database (including signal peptide).

## Phosphorylation

Protein phosphorylation is a reversible PTM where an amino acid (generally S/T/Y and to a lesser extent H) undergoes the addiction of a charged group (phosphate), possibly altering the corresponding interaction with nearby protein residues. Accordingly, phosphorylation may alter the structural conformation of a protein, causing it to become modified in its function^43^. Due to the ease with which proteins can be phosphorylated/dephosphorylated, this type of modification is a flexible mechanism for cells to respond to chemical signals and external stimuli. Phosphorylation was identified as the most frequent modification present in CAs (Figure 3(B)); in particular, 15, 14, 22, 6, 4, 2, 2, 0, 4, 8, 5, 1, 1, 6 and 6 phosphorylated amino acids were identified in CA I, CA II, CA III, CA IV, CA VA, CA VB, CA VI, CA VII, CARP VIII, CA IX, CARP X, CARP XI, CA XII, CA XIII and CA XIV, respectively. Most of these modifications occurred in the CA domain. Considering CA III, CA II and CA I as reference phosphorylated proteins, due to the occurrence of the largest number of phosphoamino acids therein, and the sequence alignment reported in Figure 2, conserved modifications with respect to other CAs were identified to occur at 13 over 22, at 10 over 14, and 11 over 15 adducted residues, respectively. At 9 positions, a conserved phosphorylation was identified to occur in at least 3 CAs; at a single position, conserved modification occurred in 5 isoforms. Regarding isoforms containing additional domains besides the CA one, in addition to the phosphorylated residues discussed above, further modifications were observed in: *i*) CA IX both in the PG domain (S102) and in the IC tail (T443, T448 and Y449); *ii*) CA XII uniquely at the C-terminal IC tail (Y342); *iii*) CA XIV in the close proximity of the TM region (T286) and at the IC tail (S325).

A careful inspection of the available three-dimensional structures of the above-reported CAs demonstrated that 67, 78, 73, 67, 50, 100, 75, 100 and 50% of the modified amino acids occurred on the molecular surface of the CA domain in CA I, CA II, CA III, CA IV, CA VI, CARP VIII, CA IX, CA XIII and CA XIV, respectively. This was expected based on the general globular characteristics of the CA domain and the post-translational nature of the considered modification, which is determined by the action of specific kinases on the folded protein. Exceptions were associated with the possible occurrence of molecular events related to the local unfolding action of kinases on CA flexible portions to be modified^44^, or the direct action of above-mentioned phosphorylating enzymes on degraded and/or unfolded molecular species^45^. In general, all phosphorylated amino acids occurred far away from the catalytic cavity. Unique exceptions were Y21 in CA I, S172 in CA II and S196 in CA IV. Indeed, these residues (in non-modified form) occurred at about 12 Å from the proton shuttle (H65 in CA I, H64 in CA II and H88 in CA IV), with a possible effect of the phosphate group on the imidazole pKa value and consequently on the rate of the catalytic reaction^46^. At present, no scientific literature is available that describes the functional role of these and above-mentioned phosphorylated amino acids present in the catalytic domain of CAs. However, the widespread representation of phosphorylation events in this enzyme family suggests that the functional importance of this modification deserves further dedicated investigations. In analogy with other kinase-regulated enzymes, phosphorylation events may determine a CA domain conformation-dependent regulation of the protein activity as result of chemical signals and external stimuli. In this context, recent studies in rainbow trout gill have shown that kinase-mediated phosphorylation of a cytosolic CA increases the corresponding enzyme activity^47^; a similar functional effect was observed in the case of thylakoid lumen CA-III from *Chlamydomonas reinhardtii*, also indicating that phosphorylation highly impacts on protein intracellular localisation^48^. On the other hand, it cannot be excluded that some of the phosphorylation events reported above for human CAs could be simply related to the specific activation of various kinases towards canonical protein targets under certain patho-physiological conditions, with the concomitant, non-functional modification of selected consensus sequences present in CAs.

It has to be emphasised that the three transmembrane CAs, namely CA IX, CA XII and CA XIV, contain phosphoamino acids in their IC tail (Figure 2). In the case of CA IX, dedicated studies described the functional role of phosphorylation at these sites. Indeed, among the three phosphorylated residues, namely T443, S448 and Y449, the first two were demonstrated to be important in modulating the enzyme catalytic activity^49^, being T443 phosphorylation critical for the enzyme activation in hypoxic cells, and S448 dephosphorylation required for the fullest activity^49^. On the other hand, Y449 modification was suggested to be involved in EGFR-induced signal transduction towards the PI3/Akt kinase pathway^50^. Finally, phosphorylation at T443 and Y449 was demonstrated to be important for the interaction of CA IX with several intracellular proteins involved in the nucleocytoplasmic transport^51^, while modification at T443, S448 and Y449 was suggested to play a critical role for the association of this carbonic anhydrase with MMP14^52^.

## Disulphide bridge formation

Most of CAs have been demonstrated to contain disulphide bridges, whose number and localisation vary among protein family members. In particular, a common intramolecular disulphide bond was observed in CA IV (C46 and C229) ^53^, CA VI (C42 and C224) ^54^, CA IX (C156 and C336) ^55^^,^^56^, CA XII (C50 and C230) ^57^, and CA XIV (C40 and C221) ^58^. This disulphide was suggested to have an important structural role in all above-mentioned enzymes, related to the stabilisation of the loop encompassing the common P–P/P-T *cis*-peptide bond (P227-T228 in CA IV). This loop contains a conserved T residue (T225 in CA IV), which was demonstrated to play a key role in CA catalytic mechanism by orienting the Zn^2+^-bound hydroxide through a hydrogen bond interaction^53^^,^^57^^,^^59^. Differently from other CAs, CA IV contains an additional intramolecular disulphide bridge, namely C24-C36, which contributes to the compactness of the protein N-terminal portion. The occurrence of two disulphide bridges in the CA IV structure was associated with the unique stability of this protein in the presence of denaturants^60^.

The disulphide bond reported above is not conserved in CA VII and CARP VIII, which instead present conserved cysteines at other positions (Figure 2). In CA VII, these amino acids, namely C56 and C180, were shown to be bridged by an intramolecular disulphide bond^61^. The observation that these cysteines, also present at the same positions in CARP VIII, occurred in their reduced form in the crystallographic structure of the latter molecule^62^ and that disulphide bonds are extremely rare in cytosolic proteins, suggested that the above-mentioned S-S bridge is not present in CA VII under physiological conditions, but it was rather an artefact generated by the oxidising conditions present during sample handling^61^. Based on these results, this disulphide was not included in Figure 2.

Finally, CA IX was also demonstrated to contain an additional intermolecular disulphide bond between two C174 residues present in contiguous CA domains. This disulphide bond stabilises dimer formation in CA IX but is not essential for protein oligomerization, since C174S mutant also occurred in dimeric state^56^. This is the unique example of the presence of an intermolecular S-S bridge in the CA family.

## S-glutathionylation

Exposure to reactive oxygen or nitrogen species is often related to disease conditions associated with redox imbalance. This condition can determine reversible oxidation of cysteine residues in target proteins, which can further proceed to an irreversible molecular damage. This undesired process can be mitigated by S-glutathionylation, a specific PTM at protein cysteines that involves the reversible addition of the tripeptide glutathione (GSH), the most abundant low-molecular-mass thiol within most cell types^63^^,^^64^. A number of findings supports an essential role of S-glutathionylation in modulating protein function and in regulating cellular signalling pathways associated with viral infection, tumour-induced apoptosis and other pathophysiological processes.

Inspection of Figure 2 reveals that S-glutathionylation occurs only in two human isoforms, namely CA III and CA VII, affecting two residues in each of them (C182 and C187 in CA III and C185 and C219 in CA VII). The features and the possible functional implications of these glutathionylation sites have been largely investigated by using several experimental approaches. In the case of CA III, original studies were carried out mainly on the mouse enzyme showing that S-gluthathionylation occurs *in vivo*^65^, and has no effect on the enzyme catalytic efficiency^66^. The analysis of the 3 D structure of S-glutathionylated CA III purified from mouse liver suggested that the intermolecular disulphide bonds between CA III reactive cysteines and glutathione were not to be ascribed to a specific recognition of the small molecule, but rather to the high reactivity of C182 and C187 and the great abundance of GSH within the cell^66^. The functional implications of CA VII S-glutathionylation were instead investigated on the human enzyme through *in vitro* studies^67^^,^^68^. Also in this case, the PTM was shown to have no effect on the enzyme catalytic efficiency and again it was associated with a high reactivity of C185 and C219^67^. These data, together with the observation that CA III and VII are found in organs and tissues characterised by a high rate of oxygen consumption, suggested that both enzymes could function as oxygen radical scavengers for protecting cells from oxidative damage^69^^,^^70^. Accordingly, CA III was shown to participate into cellular defense processes from oxidative stress, protecting cells from H_2_O_2_-induced apoptosis^71–73^, and reducing the apoptosis induced by oxidative stress in pathological conditions associated with aging, such as the degeneration of the intervertebral disc^74^. Similarly, also *in vitro* studies on CA VII revealed that cells expressing this enzyme were less sensitive to apoptosis caused by oxidative stress^68^. On the other hand, additional recent studies on CA III have suggested that this component is not polyubiquitinylated in physiological conditions (in agreement with what reported below), but protein S-glutathionylation causes reversible conformational changes that highly affects molecular susceptibility to degradation by the ubiquitin-proteasome pathway, thus unveiling a functional link between oxidative stress and the removal of damaged molecular species^75^.

## S-nitrosylation

Protein S-nitrosylation, the oxidative modification of cysteine residues by nitric oxide (NO) to form S-nitrosothiols, modifies a number of proteins, also in their activity, and provides a fundamental redox-based cellular signalling mechanism^76^^,^^77^. Differently from other PTMs, it is generally considered to be non-enzymatic and may involve multiple chemical routes for its accomplishment. In agreement with preliminary evidences reporting S-nitrosylation of CA III in rat liver^78^, this isozyme is the only protein reported to be affected by this PTM (Figure 2), which occurs at C66^79^. This residue is not accessible on the protein surface but, probably being highly reactive, can be easily reached by small-size S-nitrosylating molecules, such as NO and SNOs. It localises close to proton shuttle residue (K64); this suggests that this modification may eventually affect the enzyme activity. Accordingly, novel studies are encouraged to investigate the role of S-nitrosylation in controlling CA III catalysis, and the participation of this isozyme in redox-based cellular signalling mechanisms, as already observed for other CAs in plants^80^^,^^81^.

## O-linked glycosylation

Protein O-linked glycosylation is a PTM that involves the chemical linkage of a mono-/polysaccharide molecule to the oxygen atom of S/T residues^82^. In eukaryotes, it occurs after the protein has been synthesised, and generally takes place in the endoplasmic reticulum (ER) and Golgi apparatus. Different sugars can be introduced in the protein structure; based on their nature, they can affect the protein properties in different ways by changing corresponding stability and regulating activity. O-linked glycans have various physiological functions, such as regulation of cell trafficking in the immune system, recognition of foreign material, control of cell metabolism and provision of cartilage and tendon flexibility. Because of the many functions they have, changes in O-glycosylation are important in many diseases including cancer, diabetes and Alzheimer’s.

Figure 2 shows that O-linked glycosylation was ascertained in CA I (residues S130 and S218) and in CA IX (residues S54 and T115). In CA I, information derives from studies detecting the occurrence of O-linked N-acetylglucosamine (O-GlcNAc) in erythrocyte proteins^83^. Modification affects residues presenting a high accessibility on the molecular surface and located far from the protein active site, thus excluding any potential role in modulating enzyme catalytic activity.

In CA IX, O-linked glycosylation occurs at accessible residues present in the highly disordered PG domain^84^. Interestingly, T115 is present within a region having a high sequence similarity with the keratan sulphate attachment domain of the large aggregating proteoglycan, aggrecan^85^. Mass spectrometry experiments identified di-, tri- and tetra-saccharides containing N-acetyl-neuraminic Acid (NeuAc) or N-glycolylneuraminic acid (NeuGc), and having or not a sulphate moiety, which were O-linked at this site^55^. Some of these oligosaccharides highly resemble the keratan sulphate unit that was already described to occur in the proteoglycan domain of other proteins involved in cell adhesion processes and tumour progression^86^. Studies aimed at elucidating the functional role of this modification on CA IX are currently not available. On the other hand, more complex O-linked glycosaminoglycan (GAG)-like structures^87^ were detected at S54, which accounted for a mass shift of 20–50 kDa with respect to the non-modified protein^88^. Their nature was ascertained as chondroitin and heparan sulphate simply based on the specificity of enzymes (chondroitin ABC lyase and heparanase III) used for protein digestion. Novel and more informative studies are requested to fully characterise this CA IX modification.

Both O-linked glycan modification sites in CA IX occur in the PG domain. This domain has been reported to have a high conformational flexibility and to be involved in partner recognition^84^. This finding and the nature of ascertained PTMs, generally involved in mediating molecular interactions, envisaged a functional role for these modifications. In agreement with this hypothesis, Belting and co-workers recently demonstrated an important consequence of GAG conjugation at S54 in CA IX^88^. In particular, authors showed that cancer cell internalisation of CA IX is negatively regulated by this PTM, and occurs through a molecular mechanisms unrelated to dimer formation and/or enzymatic activity. More importantly, they demonstrated that pharmacological inhibition of GAG biosynthesis potentiates the internalisation and the cytotoxic activity of an antibody-drug conjugate targeting CA IX, disclosing important scenarios for the future development of targeted cancer treatments.

## N-linked glycosylation

N-linked glycosylation is an enzyme-catalysed process involving the attachment of an oligosaccharide (also referred to as glycan) to the amide nitrogen of protein N residues^89^. The attachment of the glycan chain to the protein requires the enzymatic recognition of a consensus N-X-S/T sequence. In eukaryotes, the biosynthesis of N-linked glycoproteins starts in the ER, continues in the Golgi and ends at the plasma membrane, where they are either secreted or become embedded in the plasma membrane. The nature of the attached N-linked glycans is determined by the protein and the cell in which it is expressed. Depending on the nature of the various monosaccharides adducted at the common N-linked pentasaccharide (mannose_3_N-acetylglucosamine_2_ - Man_3_GlcNAc_2_), different oligomannose, complex, and hybrid structures occur in glycoproteins. These structures mediate many protein properties, such as corresponding conformation, folding, solubility and antigenicity as well as cell-matrix and cell-cell interactions^90^. Because of their impact in various biological processes, protein N-linked glycans can be used as a diagnostic marker for the diagnosis and monitoring of various chronic diseases and cancers.

Various N-linked glycosylation sites were identified in the secreted CA VI (N67 and N256), the cytosolic CARP XI (N118 and N260) and the membrane-bound isoforms CA IX (N346), CA XII (N80) and CA XIV (N213) (Figure 2). A careful analysis of the available crystallographic structures (CA VI, CA IX, CA XII and CA XIV) highlighted that all above-reported modified amino acids occur on the molecular surface and are far away from the corresponding active sites. This parallels with information on chloroplast CA I from *Arabidopsis thaliana*, which contains five N-linked glycosylation sites pointing towards the solvent and occurring not close to catalytic residues^91^; in this case, N-glycosylation was proved to be essential for correct protein folding, ER secretion and subcellular localisation. Below, we summarise the available data on the possible functional role of the ascertained N-linked oligosaccharide structures in above-mentioned human CAs.

N-glycosylation of human CA VI was firstly described in 1998 by Thatcher and co-workers, who showed that two out of the three putative glycosylation sites, namely N67 and N256, were actually modified with glycans containing GlcNAc, galactose (Gal), Man and fucose (Fuc) units, which were interior to di-, tri- and tetra-sialyated termini^92^. In this study, no information on the functional role of these PTMs was provided, which was on the contrary suggested in a parallel investigation on the corresponding bovine enzyme. Indeed, Hooper and co-workers showed that the glycosylation features of the bovine CA VI are dependent on the tissue in which the protein is expressed^93^. In particular, they demonstrated that the enzyme from the submaxillary gland mainly contains N-linked oligosaccharides terminating with N-acetylgalactose-sulphate (GalNAc-4-SO_4_) units, whereas the counterpart from the parotid gland has terminal non-sulfated GalNac moieties. Based on this evidence, the authors suggested a different biological role for the submaxillary and parotid enzymes, also hypothesising that receptors with a different specificity for GalNAc-4-SO_4_ or GalNAc may be eventually present in different areas of the oral mucosa to selectively immobilise these CA VI forms. The differential immobilisation of the submaxillary and parotid enzyme forms in specific regions of the mouth should represent a physiological mechanism for locally regulating the oral pH^93^. In this context, it is worth noting that the addition of GalNAc to N-linked oligosaccharides by β1,4-N-acetylgalactosaminyltransferases requires a specific sequence present in bovine CA VI, which corresponds to a 19-residue peptide at the protein C-terminus presenting a high content of basic amino acids and an α-helical structure^94^. This sequence is not conserved in human CA VI, highlighting the need of further studies to identify the eventual presence of GalNac also in the human enzyme as well as of the above-mentioned differences in corresponding submaxillary and parotid enzyme forms.

On the other hand, detailed mass spectrometric studies on membrane-associated human CA IX expressed in baculovirus-insect system and murine cells demonstrated the occurrence of a N-linked glycosylation site (N346) in the catalytic domain of both products, which was modified by high mannose-type glycans^55^. In the case of the murine expression system, N-linked hybrid-type structures were also detected. Since this isozyme is overexpressed in several types of cancer^5^^,^^95^^,^^96^, a more detailed characterisation of the post-translational processes modifying the final protein structure is necessary to investigate the role of N-linked glycans and their eventual changes during malignant transformation.

Although human CA XII contains three putative N-glycosylation sites (N28, N80 and N162)^97^, our search in PTM databases revealed an ascertained modification only at N80. However, a dedicated investigation by Hong and co-workers indicated the occurrence of N-linked glycosylation at N28 and N80^97^. Although this study did not provide information on the nature of the linked glycan structures, a clear correlation between the occurrence of N-glycosylation and protein subcellular localisation was highlighted. Indeed, the E143K CA XII mutant, which is responsible for an autosomal recessive form of salt wasting^98–100^, presented an altered modification at N28 and N80, and consequently was retained in the ER, differently from the native enzyme that was expressed in the basolateral membrane^97^.

Only one N-linked glycosylation site was identified in CA XIV, namely N213. This site was initially predicted by Fujikawa and co-workers^101^ and subsequently confirmed by the crystal structure determination of the human enzyme expressed in baculovirus-insect cell system^58^. In the latter study, analysis of the electron density maps allowed the clear identification only of the glycan moieties present in the pentasaccharide core N-linked to N213. Further experiments are necessary to provide additional information on the nature of the additional glycans present therein as well as on their possible functional role.

At present, no information is available on the structure of the N-linked glycans and corresponding function of the two glycosylation sites identified in CARP XI. All results reported above underline the need of novel dedicated investigations to fully characterise N-linked glycans in human CAs and to shed light on their possible impact of corresponding protein function.

## Non-enzymatic glycation

Non-enzymatic protein glycation is an irreversible PTM affecting K and R residues as well as N-terminal amino acids, which is generally initiated by the condensation of the carbonyl group of reducing sugars with the amino group of proteins. At first, it generates a Schiff base (aldimine) adduct that is unstable and can rearrange via an enamine intermediate to generate a 1-amino-1-deoxy-2-ketose (ketoamine) product, also known as the Amadori compound. In the case of glucose, this reaction yields to N^ε^-(1-deoxy-D-fructos-1-yl)-lysine. Glycated proteins can either further react to form advanced glycation endproducts (AGEs), which contain modified K and R residues, or react directly with sugar-derived dicarbonyl compounds also to form AGEs^102^. In human, this process generally occurs under patho-physiological conditions generally associated with a hyperglycaemic status, such as diabetes, but it has also been observed in the course of neurodegenerative and cardiovascular diseases, and aging. Extensive non-enzymatic glycation can alter the three-dimensional structure of a protein, with possible effects on its functional properties^103–105^.

Non-enzymatic glycation was identified as a frequent modification in CAs, and especially in those isoforms highly abundant in biological fluids^106–108^. In particular, 10, 9 and 2 glycated amino acids were identified in CA I, CA II and CA III. Considering these CAs and the sequence alignment reported in Figure 2, in two cases glycation occurred at the same conserved K residue in CA I and CA II. Often it happened at K residues that also underwent acetylation and/or ubiquitinylation in above-mentioned proteins.

A focussed analysis of the crystallographic structure of the above-reported CAs suggested that all above-reported non-enzymatic glycated amino acids occurred on the molecular surface of CA I, CA II and CA III, respectively. These residues are greatly accessible and thus can easily react with glucose molecules. At the same time, collected data excluded the occurrence in CAs of glycation events involving internal lysines with a great reactivity deriving from either the close proximity of specific amino acids in the primary and/or the tertiary structure favouring formation of the Amadori compound^106^, or local unfolding events in partially modified CAs. In general, all glycated amino acids were not present in the enzyme catalytic cavity. Unique exception was K64 in CA III, which is suggested to be the proton shuttle, with possible, significant consequences on the rate of the catalytic reaction^46^.

In a functional context, worth-mentioning is the extensive glycation of CA I, CA II and CA III, detected in the plasma and erythrocytes from diabetic subjects^106–108^. When glycated CA I was purified from erythrocytes of diabetic patients, it was demonstrated to present a specific enzymatic activity that was approximately 40% of that of the non-modified enzyme from normal individuals^109^. Other studies on CA II demonstrated that extensive protein glycation is accompanied with a significant conformational change and a reduced enzymatic activity^110^. Above-mentioned investigations suggested that changes in general CA activity in human erythrocytes due to protein glycation can be considered among the initial steps of altered metabolism in diabetes^111^. These findings underline the functional impact of this modification on CAs in diabetes and provide evidences on the need of further dedicated studies on glycated CAs in physiological and disease conditions for which the formation of AGEs is a patho-physiological hallmark, e.g. aging as well as neurodegenerative and cardiovascular diseases.

## Acetylation

Protein acetylation is one of the most represented PTMs in the cell. In reactions catalysed by various N-terminal and lysine acetyltransferases, the acetyl group of the metabolite acetyl-coenzyme A is co- or post-translationally adducted to either the α-amino group of proteins or to the ε-amino group of corresponding K residues^112^. In the latter case, the reaction can be reverted by deacetylases through tightly-regulated and metabolism-dependent mechanisms. The interplay between acetylation and deacetylation is crucial for many important cellular processes. Since it converts a positively-charged group into a neutral moiety, acetylation can alter the structural conformation of a protein as well as its capability to interact with other molecules, with a possible impact on corresponding function. Accordingly, acetylation can regulate a number of metabolic and physiological processes, with a profound impact on cell and organism life.

Inspection of Figure 2 reveals that acetylation occurs in CA I (at the N-terminus), CA II (at the N-terminus, K18, K39 and K113), CA III (at the N-terminus and K126) and CA XII (at K194). Some of these K residues are also affected by ubiquitinylation. In all cases, acetylation was verified occurring at residues well exposed to the solvent and far away from the active site, excluding any possible consequent effect on the enzyme catalytic activity. At the present, no information is available on the functional role of acetylation at above-mentioned residues. On the other hand, Whitesides and co-workers published interesting studies concerning the effects of acetylation in CAs using bovine CA II as model protein; the latter contains 18 positively charged K residues on its surface^113,114^. These investigations demonstrated that acetylation at these K residues does not alter the 3 D structure, the ability to refold as well as the rate of refolding from solutions of this enzyme containing SDS^113^^,^^114^, and suggested that acetylation might play a critical role in protein-protein interaction processes by increasing the participation of polar residues (mainly acetylated K) in corresponding molecular interfaces, and decreasing the contribution of non-polar residues in these contact regions^113^. In a similar way, although the acetylated sites in CA I, II, III and XII isoforms are less numerous than those artificially introduced in bovine CA II, it can be hypothesised that *in vivo* acetylation processes involving CAs should play a significant role in modulating corresponding molecular interactions within the cell.

## Ubiquitinylation

Protein ubiquitinylation is a PTM in which the polypeptide ubiquitin is covalently added to K residues^115^. This reaction is catalysed by a number of E3 ligases, each of which transfers ubiquitin to corresponding protein targets. Depending on the addition of poly- or mono-ubiquitin chain, different effects are exerted on target proteins. In particular, addiction of ubiquitin polymerised through K48 residue acts as a signal targeting proteins for proteasomal degradation. Conversely, addiction of a poly-ubiquitin chain linked through other K residues can alter protein subcellular localisation or interactions. The latter effects are also exerted by mono-ubiquitinylation on target proteins. Ubiquitinylation is often targeted to newly synthesised proteins, which are highly susceptible to misfolding and aggregation. This PTM-based quality control ensures that misfolded proteins undergo proteasomal degradation. Ubiquitinylation sites on nascent misfolded proteins act as valuable biomarkers of cellular states, in which protein synthesis and/or folding has been disrupted, as observed in Alzheimer’s and Parkinson’s disease^115^. In response to signalling or other pathways, properly folded proteins can be also subjected to regulatory ubiquitinylation, which can influence protein functions and localisation.

Lysine ubiquitinylation was identified to occur in various CAs; in particular, 6, 1, 3, 3 and 2 ubiquitinylated amino acids were assigned in CA II, CA VA, CA VB, CARP VIII and CA XII, respectively. Some of these K residues were also affected by acetylation and non-enzymatic glycation, highlighting the occurrence of a putative signal modulation at these sites. A careful inspection of modified amino acids present in the available three-dimensional structure of above-mentioned CAs (CA II, CARP VIII e CA XII) suggested that all ubiquitin adduction events occurred on the molecular surface, and at sites not present in protein portions related to the enzyme catalytic function. In trans-membrane CA XII, modification occurred in the IC tail, at amino acids that may eventually affect protein interaction with intracellular molecular binding partners, similarly to what phosphorylation does in CA IX (see above); further studies are encouraged in a next future to address this issue.

## Glycosylphosphatidylinositol-anchoring

Glycosylphosphatidylinositol (GPI) adduction is a complex modification process that occurs during the secretory pathway of certain proteins, allowing their final anchoring to the cellular membrane even if they do not contain a transmembrane domain. Genes encoding GPI-modified proteins generally contain two signal sequences in their primary translation product, namely an N-terminal signal sequence for ER targeting and a C-terminal sequence motif guiding the covalent attachment of the GPI anchor; both segments are removed in the mature membrane-bound product. The deletion of the first sequence generally occurs in ER^116^, while that of the latter is synchronous to the GPI transamidase-catalysed attachment of the GPI anchor^116^^,^^117^.

Figure 2 shows that CA IV is the only isoform presenting a GPI-linked residue, namely S284. This amino acid is conserved in the isozyme counterpart from other vertebrates (data not shown), and is essential for protein anchoring to the plasma membrane of specific epithelial and endothelial cells. In fact, combined biochemical and mutagenesis experiments demonstrated that S284F replacement prevents protein GPI anchoring and cell surface expression, results in polypeptide retention within ER, and induces a rapid enzyme degradation^116^. These studies also showed that removal of the protein C-terminal hydrophobic domain is also fundamental for GPI-anchoring; indeed, the C-terminal truncated G285X mutant was secreted into the medium, while the G285F one still underwent GPI anchoring, was expressed on cell surface and was fully active^116^.

CA IV has been widely characterised for its kinetic properties, showing that it is a very efficient catalyst for the hydration of carbon dioxide (K_cat_/K_M_ value of 5.1 × 107 M^−1^ s^−1^)^118^^,^^119^. Interestingly, the GPI-anchoring does not have any effect on catalytic activity. Indeed, the truncated G285X mutant showed the same specific activity of the GPI-anchored enzyme^116^. This finding is in good agreement with structural studies providing a model of CA IV anchored to the cell membrane^53^, which demonstrated that the site of the GPI-anchoring is very far from the active site, and does not influence neither the substrate binding nor the catalytic reaction. Conversely, it occurs in a molecular region presenting a large electropositive surface potential, which stabilises protein interaction with the negatively charged phosphate groups of the phospholipid bilayer present in membranes^53^.

## Methylation

Protein methylation is a PTM featuring the addition of methyl groups to proteins. Generally, it affects the side-chain of R and K residues^120^. The former can be methylated once (monomethylated R) or twice (dimethylated R) by different classes of protein R methyltransferases (PRMTs). The latter can be methylated once, twice, or three times by protein K methyltransferases (PKMTs). Similarly to phosphorylation, methylation can introduce a net charge on the amino acid side chain, possibly affecting the interaction of proteins. Thus, this process can regulate different biological processes.

Methylation was detected at R229 in CA VII and R328 in CARP XI. In the first case, this modification occurs on the protein surface and far away from the active site of the enzyme, thus excluding any possible effect on corresponding catalytic properties. Similar considerations cannot be done for CARP XI, since its crystallographic structure is not yet available.

## Conclusions

By catalysing the reversible hydration of carbon dioxide to bicarbonate ion and proton, CAs are involved in a number of physiological processes related to respiration, carbon dioxide transport, pH and electrolyte homeostasis, and biosynthetic reactions. Their abnormal expression and/or activity have been related to a number of pathologies and diseases, including glaucoma, edema, obesity and cancer^1^^,^^2^. Accordingly, a number of inhibitors belonging to different molecular types has been proposed with the aim to limit and control their catalytic activity, sometimes according to an isozyme-directed approach, whose promising application as pharmacological agents sometimes reached phase I-III clinical trials, and/or finally allowed the production of well-established drugs^1^^,^^2^. Notwithstanding the abundant literature on biochemical, functional and structural aspects of CAs, limited information is available on corresponding PTMs, which generally derived from specific isozyme-focussed biochemical studies. In the last decade, the development of proteomic technologies allowed performing PTM-centred studies in which additional modification information on CAs was determined, which is generally recorded in the supplementary material of published articles and in deriving publicly available modification databases, whose relevance often escaped proper attention. The widespread occurrence of modification events in all CAs, often detected at conserved amino acids, suggests that the functional importance of these modifications still deserves further dedicated studies, which have to be performed by systematic investigations on protein mutants generated by site-directed mutagenesis, taking advantage of integrated experimental approaches. Thus, the discovery that all CAs are subject of phosphorylation, non-enzymatic glycation, N-glycosylation, O-glycosylation, S-glutathionylation, S-nitrosylation, disulphide-formation, acetylation, ubiquitinylation, methylation, and GPI-anchoring, their punctual description (as described in this study), and the recent development of specific CA isoform-selective drugs open novel broad scenarios of investigation to better understand the relevance of these proteins, how PTMs modulate their biological functions, and whether their biological properties can be pharmacologically manipulated according to still-unexplored therapeutic purposes.
